# Reactions of 5-Indolizyl Lithium Compounds with Some Bielectrophiles

**DOI:** 10.3390/molecules22040661

**Published:** 2017-04-20

**Authors:** Sergey A. Rzhevskii, Victor B. Rybakov, Victor N. Khrustalev, Eugene V. Babaev

**Affiliations:** 1 Life Sciences Center, Moscow Institute of Physics and Technology, Institutskii per. 9/7, Dolgoprudniy, Moscow Region 141701, Russia; rs89@rambler.ru; 2 Chemistry Department, Moscow State University, Moscow 119991, Russia; rybakov20021@yandex.ru; 3 Peoples’ Friendship University of Russia (RUDN University), Miklukho-Maklaya str. 6, Moscow 117198, Russia; vnkhrustalev@gmail.com

**Keywords:** indolizine, 5-indolizyl lithium, reactivity, bielectrophilic reagents

## Abstract

Indolizyl-5-lithium anions react with succinic and phtalic anhidrides giving 1,4-keto acids, with oxallyl chloride giving 1,2-diketone, and with ethyl pyruvate giving 1,2-hydroxyacid. However, with α-halocarbonyl compounds, they react in different ways, forming the products of selective bromination at C-5 (with α-bromo ketones and esters of α-bromo acids) and 5-chloroacetyl indolizines.

## 1. Introduction

Indolizine is an important heterocycle, and its derivatives show interesting photophysical (fluorescence) and biological properties [1,2,3,4,5,6]. It is well known that electrophilic reagents react with indolizines at position 3 and/or 1 [7,8,9]. However, indolizines are easily deprotonated at C-5 under the action of BuLi, and the use of an intermediately lithiated indolizines leads to an attack of electrophiles on the carbanionic center C-5. In the literature, there are few reactions that allow such groups as carboxyl, acyl, methyl, halogen or trimethylsilyl [10,11] to enter the carbanionic center at position 5, as well as subsequent Suzuki [12] and Sonogashira [13] coupling. However, the reactions with polyfunctional electrophiles are not described, and the aim of this work is to analyze the reactions of 5-indolizyl lithium with some bielectrophilic reagents. In this case, both C-5 and C-3 positions in the indolizyl anion are nucleophilic, and the entire structure would resemble a sort of 1,3-binucleophile that may undergo cyclization to a tricycle.

## 2. Results and Discussion

For our studies, we selected indolizines containing phenyl (**1a**) or *tert*-butyl (**1b**) group at position 2. Just for these substrates, the lithiation reaction, as well as further reactions with electrophiles, were studied [10,11]. In addition, 2-*tert*-butyl-indolizines are much more resistant to oxidation compared to other 2-alkyl indolizines, and most of the 2-phenyl substituted indolizines are crystalline compounds.

Lithiation of the compounds **1a** and **1b** was carried out by the technique developed in our laboratory. In the standard method [10], it is recommended to pour a stream of electrophile solution into the indolizyl anion. In order to realize the slow addition of a carbanion to the excess of electrophile, its solution was pressed through a curved metal needle directly into the electrophile solution under a pressure of argon. Alternatively, the carbanion solution could be moved to a funnel and then added to the electrophile.

### 2.1. The Reactions with Succinic and Phtalic Anhydrides

**Scheme 1:**
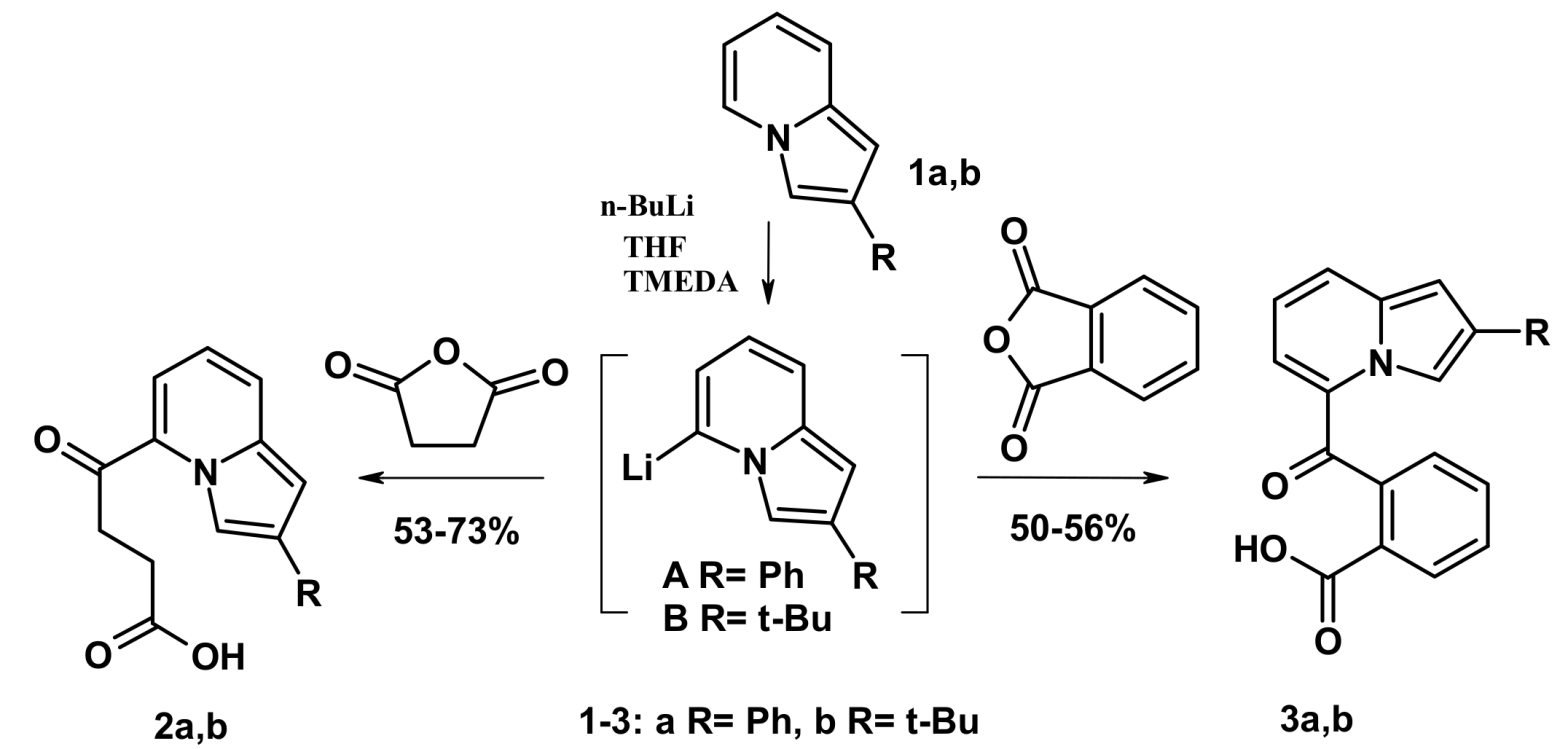
Reaction of indolizyl lithium compounds with the succinic and phtalic anhydrides.

When indolizyl lithium derivatives **A** and **B** (from the compounds **1a** and **1b** respectively) reacted with succinic or phtalic anhydride, the color of the reaction mixture was changed to bright yellow or red. After treatment with aqueous NH_4_Cl and chromatography on silica gel (CHCl_3_-CH_3_OH, 10:1), new crystalline substances were isolated, Scheme 1. The structures of the thus obtained compounds **2a** and **2b** (yellow) and **3a** and **3b** (red) corresponded to the products of opening of these cyclic anhydrides by indolizyl anions leading to keto-acids.

**Figure 1:**
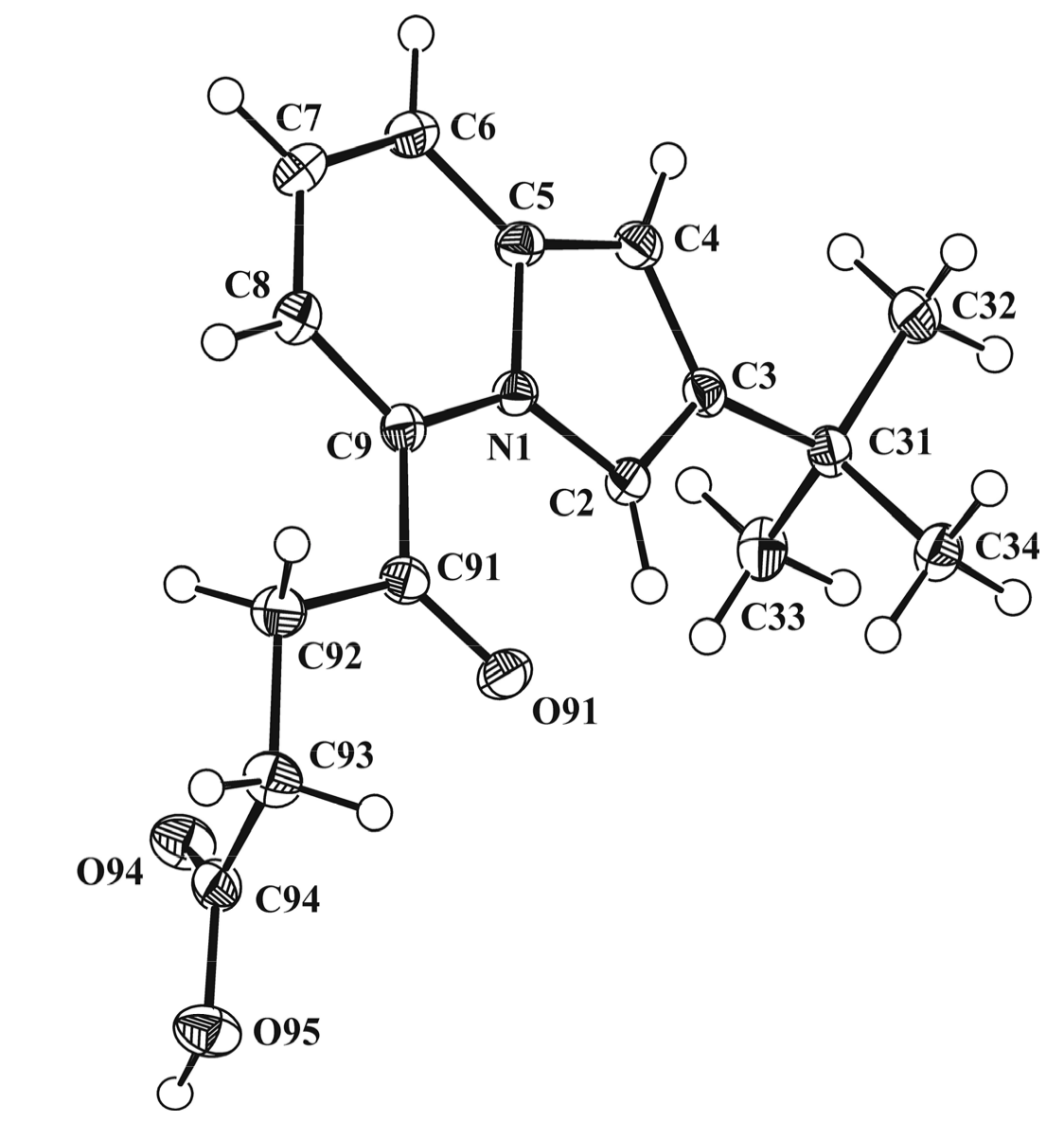
X-ray data for compound **2b**.

The elemental composition of the compound **2a** corresponded to hemi-hydrate, and that of compounds **3a** and **3b** corresponded to hydrates; the water could not be removed by vacuum heating. The structure of molecule **2b** was proven by X-ray data, Figure 1. The mass spectra of compounds **3a** and **2b** had the expected peaks of molecular ions. The color of compounds **3a** and **2b** was caused by long-wave maximum at ~450 nm.

In the ^1^H-NMR spectra of compounds **2** and **3**, the doublet of proton H-5 (shifted most downfield in the spectra of starting materials **1**) disappeared, and the signals of keto-acid appeared in the aliphatic (or aromatic) region. The characteristic feature of the spectra of all compounds **2** and **3** was the presence of the isolated and most downfield singlet H-3 in the area of 8.8–9.2 ppm which resulted from the *peri*-effect of the magnetically anisotropic 5-acyl group. This effect was observed for other 5-acyl indolizines (formyl- and benzoyl derivatives [11]). Furthermore, 3- and 8-acyl indolizines behaved similarly [14] with the influence on *peri*-positions H-5 or H-1. Thus, indolizyl lithium derivatives reacted with succinic or phtalic anhydride, forming previously unknown ketoacids **2** and **3**.

### 2.2. The Direction of Protonation

**Scheme 2:**
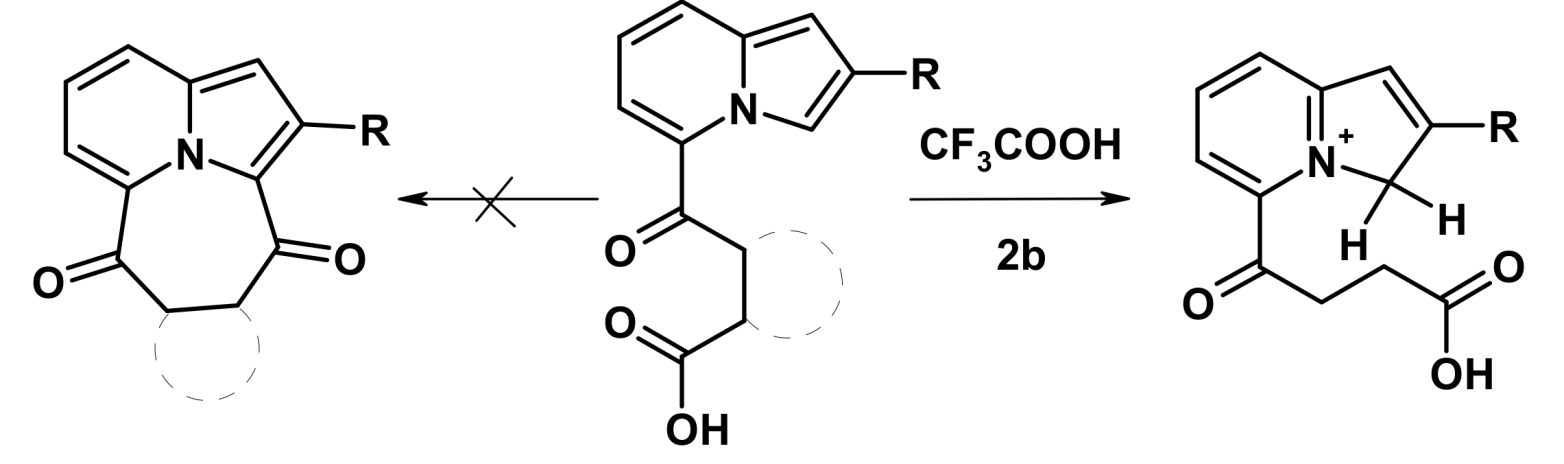
Hypothetical (**left**) and real (**right**) direction of action of an acid on indolizines.

The location of the electrophilic carboxyl function in the compounds **2** and **3** near the electron-excessive atom C-3 of the pyrrole moiety of indolizine allowed the possibility of ring closure to be expected. It may occur under the proper activation of the COOH group leading to intramolecular acylation of the indolizine ring (Scheme 2).

Meanwhile, all our efforts to encourage such a cyclization (by dissolving compounds **2** and **3** in protonic acids of different strength) have not led to the desired result. While such agents are often used to assist intermolecular cyclizations of indolizines, in our experiments we isolated only unchanged starting materials. The reason could be the well-known fact that the indolizine nucleus itself is the base; mineral acids protonate the indolizine moiety at C-3.

We registered the ^1^H-NMR spectrum of compound **2b** in CF_3_COOH and observed that indolizine was entirely protonated at C-3, Scheme 2. In the ^1^H-NMR spectrum, there was a singlet of double intensity of H-3 at 5.8 ppm, while the rest of the signals were shifted downfield. The literature data of the spectra of 1- and 3-CH_2_-indolizyl cations [14,15,16] indicate that the position of a singlet of the CH_2_ group at δ > 5 ppm is clear proof of protonation at position 3. Since the protonated atom C-3 is excluded from the π-excessive heterocyclic system, its subsequent acylation becomes impossible. It is worth noting that, in the mass spectra of compounds **2b** and **3a,** there is no peak [M − 18]^+^ corresponding to the loss of water molecules through intramolecular cyclizations of the molecular ion. This may indirectly indicate a low tendency of the compounds **2** and **3** to transform in tricycles. Possible closure of the seven-membered ring is generally unfavorable due to steric reasons.

### 2.3. The Reaction with Oxallyl Chloride and Ethyl Pyruvate

**Scheme 3:**
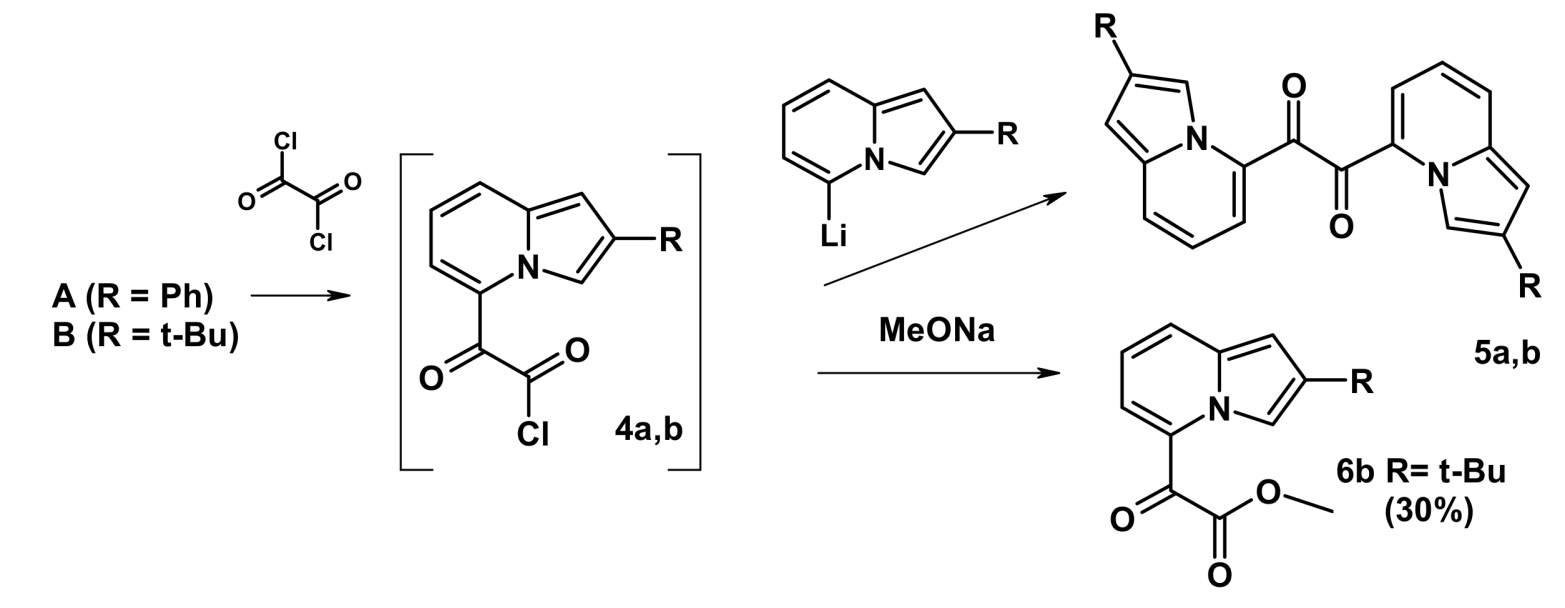
Reaction of indolizyl lithium derivatives with oxallyl chloride.

**Figure 2:**
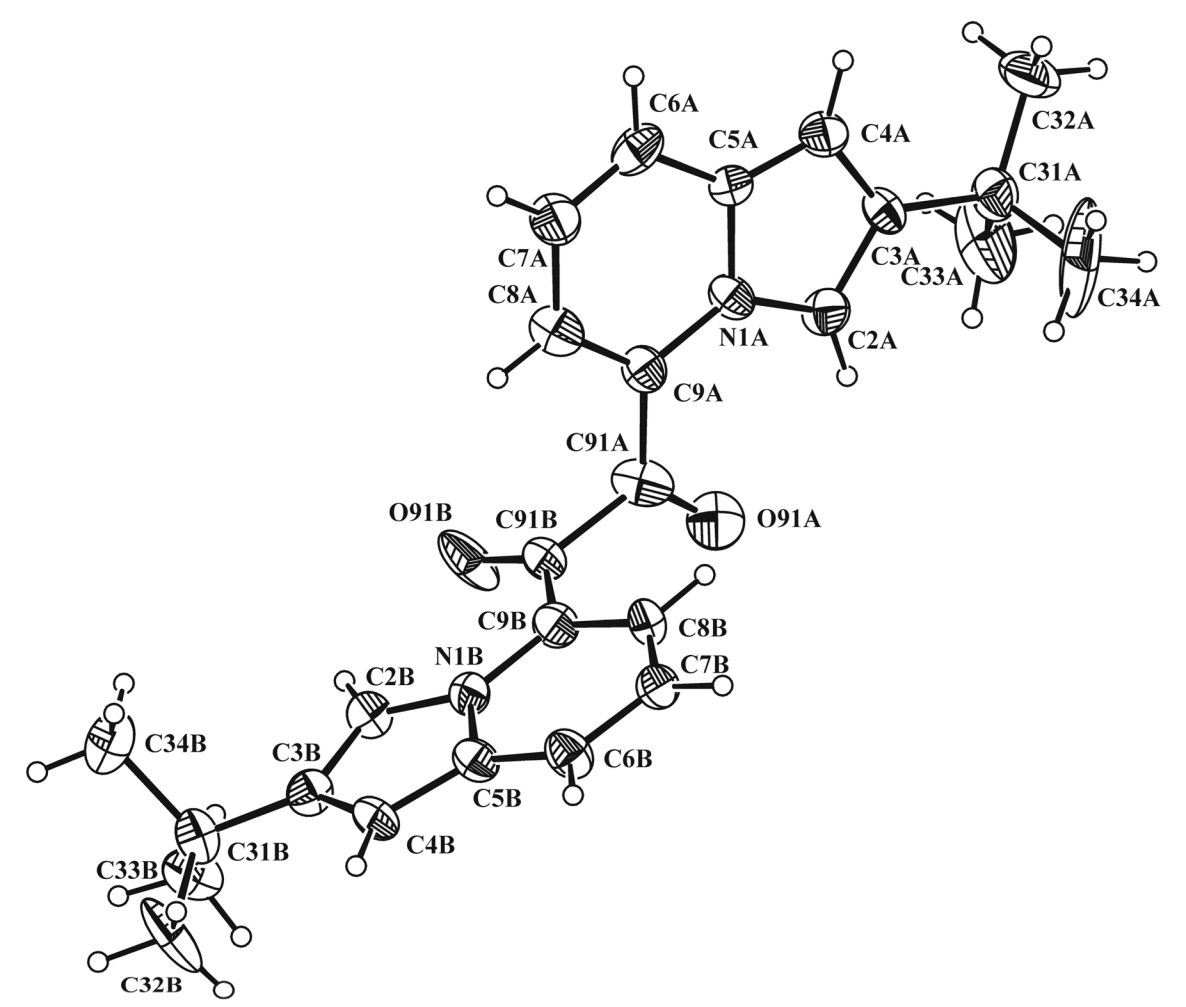
X-ray data for compound **5b**.

In the next stage, we decided to shift from the case of 1,4-bielectrophiles to the extreme case of 1,2-bielectrophiles, i.e., derivatives of 1,2-dicarboxylic acid, namely, its *bis*-acid chloride. When adding oxallyl chloride to lithium-organic derivatives **B** (from indolizine **1b**), the reaction mixture was colored intensively red. After purification, a red crystalline solid was obtained, the ^1^H-NMR spectrum of which was very similar to the above-described 5-acyl derivatives **2** and **3**. Thus, in the spectrum, the signal of proton H-5 was absent, and the proton H-3 resonated at the weak field at 8.96 ppm. Initially, we assumed that the structure of the compound met the structure of acid chloride **4b**. Meanwhile, a study of the mass spectrum (LCMS) indicated the presence of the peak [M]^+^ = 401, corresponding to the diketone **5b**, Scheme 3. This structure was also confirmed by elemental analysis data. Finally, the X-ray data (Figure 2) confirmed that the structure of the resulting product **5b** really contained two pieces of indolizine and diketone.

A similar result was obtained for 2-phenyl indolizine **1a**. In this case, an insoluble residue precipitated from the reaction mixture. Its mass spectrum (direct input) showed the presence of a peak [M]^+^ = 283 (which corresponded to acid chloride **4a**) and a peak of the dimeric structure **5a** ([M]^+^ = 440).

Using indolizine **1b**, we conducted the same experiment, slowly adding (via a curved metallic needle) solution of indolizyl lithium **B** to oxallyl chloride. The solution had the expected red color, but analysis of the reaction mixture by TLC showed that the main product (with low *R*_f_) decomposed during chromatography. To prove that, in this experiment, the acid chloride **4b** was really formed, the reaction mixture was decomposed with a solution of MeONa. In this case, a stable crystalline solid was formed, the ^1^H-NMR spectrum of which greatly resembled that of diketone **5b**, but in the aliphatic region the signal of the MeO group was presented. This spectrum was fully consistent with the structure of the ester **6b**, Scheme 3.

**Scheme 4:**
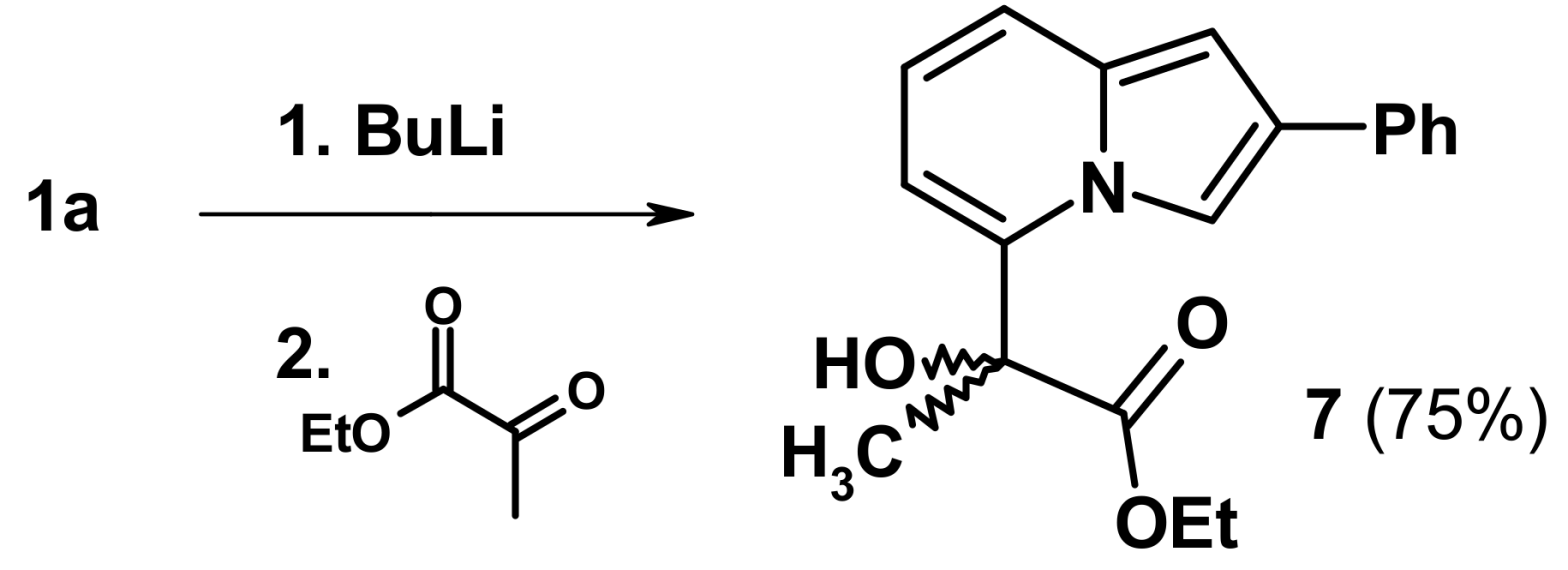
Reaction of the indolizyl lithium compound (**A**) with ethyl pyruvate leading to compound **7**.

To expand the range of 1,2-bielectrophiles, we studied the reaction of lithium derivative **A** with ethyl pyruvate. According to the ^1^H-NMR spectrum of the product, the ester group was observed, indicating that the attack of the carbanion was directed at the acetyl moiety of ethyl pyruvate, forming the hydroxy acid ester **7**, Scheme 4. Since, in this case, there was a tertiary alcohol at position 5, the *peri*-effect was not observed in the spectrum, and the proton H-3 resonated at higher fields (7.85 ppm). An anticipated feature of the NMR spectrum of the compound **7** was the complex multiplicity of the signal of the methylene unit of the ethoxycarbonyl group, due to the influence of diastereotopic methyl and hydroxy groups on the chiral carbon atom.

### 2.4. The Reaction with Phenacyl Bromide and Haloacetic Acid Esters

**Scheme 5:**
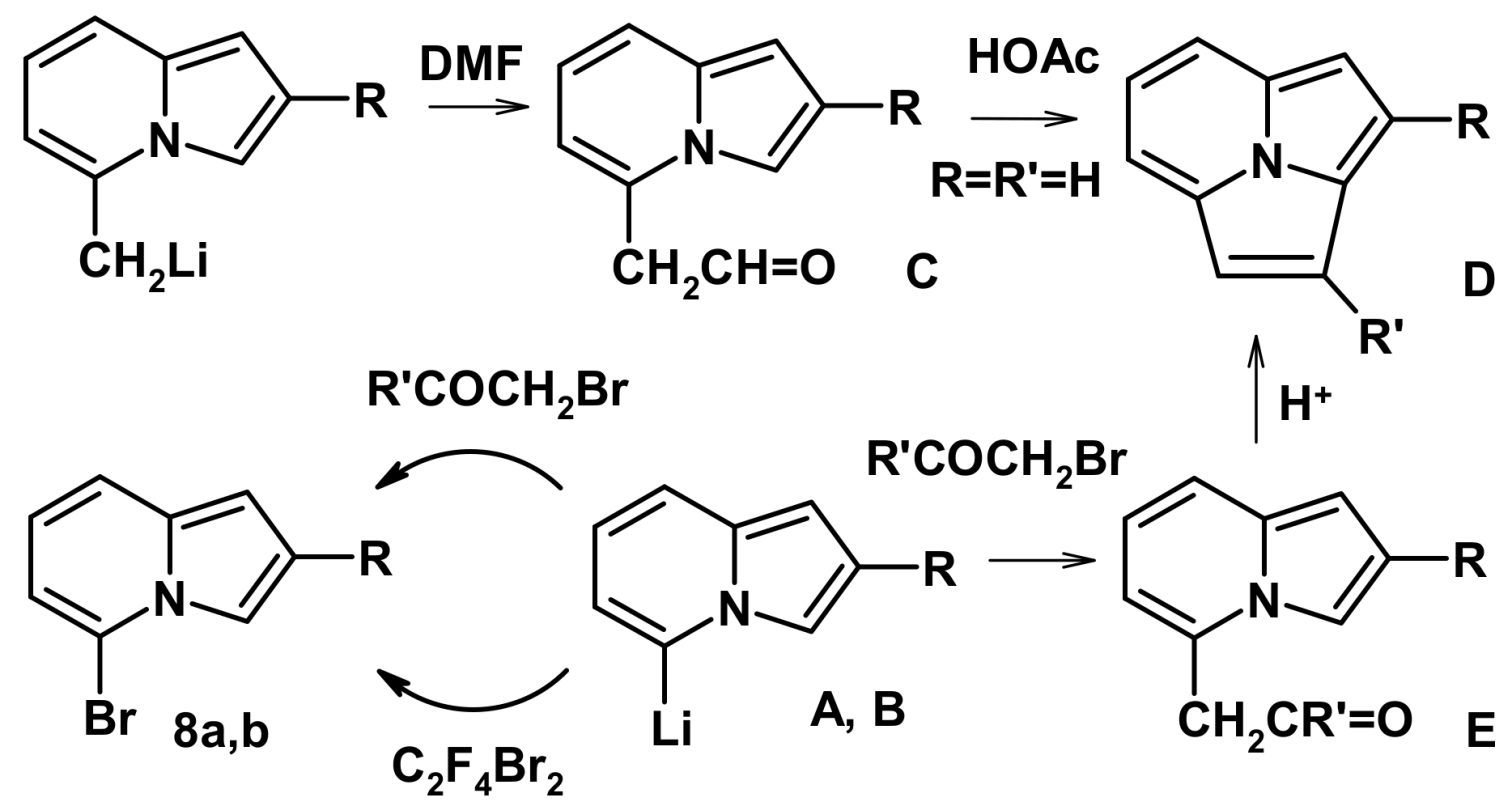
Pathways of reactions of indolizines with phenacyl bromides.

As was shown by Boekelheide in 1951 [17], the lithiation of 5-methyl indolizine followed by reaction with DMF led, through the intermediate **C**, to the formation of a cycle[3.2.2] azine **D** (Scheme 5). We expected that phenacyl bromide in reaction with indolizyl lithium derivatives **A** and **B** may lead to a structurally analogous intermediate **E**, which is also capable (under the action of acids) of closing the ring of cyclazine **D**.

The addition of phenacyl bromide to derivatives **A** and **B** resulted in compounds **8a** and **8b** (Scheme 5). In the ^1^H-NMR spectra, the doublet H-5 of starting indolizines was absent, but the signals of the corresponding phenacyl group were also not observed. We supposed that the bromination at position C-5 happened instead of alkylation. Comparison of the m.p. (°C) of compound **8a** with the literature data [11] (as well as the data on the ^1^H-NMR spectra of both substances) confirmed this assumption. Finally, the structure of indolizines **8** was proven by direct bromination of indolizyl lithium derivatives **A** and **B** by mild brominating agent (C_2_F_4_Br) according to the method described in the literature [11]. Although there are examples where phenacyl bromides may act as electrophilic brominating agents, such processes, however, generally occur in an environment of strong acids. Meanwhile, we fail to find literary examples (and of course, the mechanism) of the bromination of carbanions (or lithium-organic compounds) using alpha-bromoketones. The reason for the observed transformation could lie in the comparable electronegativity of the bromine and CH_2_COPh group, which underwent cleavage to Br^+^ and carbanion at certain conditions.

**Scheme 6:**
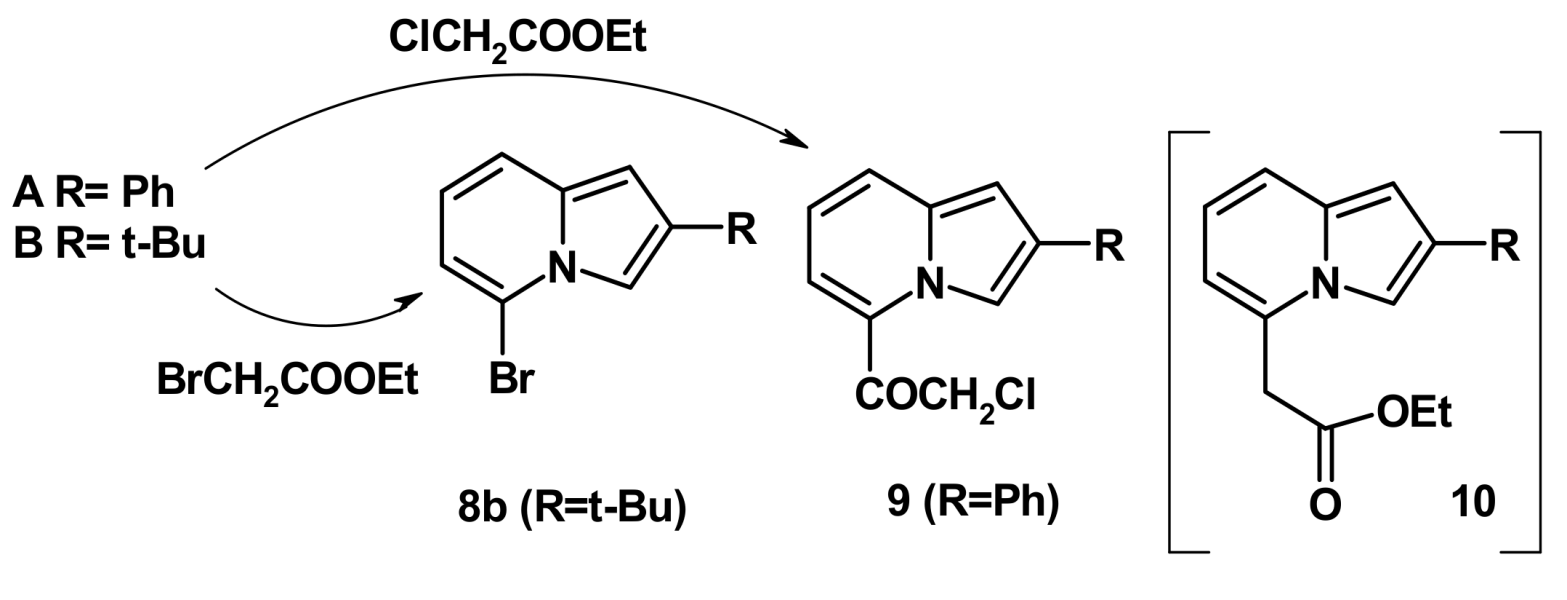
Reactions of indolizines with haloacetic acid esters.

The reaction of ethyl bromoacetate with the lithium derivative **B** also resulted in the formation of 5-bromoindolizine **8b**. Hoping that a more electronegative chlorine atom would show lower electrophilicity as compared with bromine, we attempted to involve ethyl chloroacetate in reaction with the lithium derivative **A**, Scheme 6.

The reaction product, however, according to the ^1^H-NMR spectra, greatly resembled that of 5-acyl derivatives (**2**, **3**, **5**, **6**). The signal of proton H-5 was absent in the spectrum, and the singlet H-3 was downfield (9.44 ppm). Signals of the ethoxycarbonyl group (expected in the case of the alkylation of ethyl chloroacetate at C-5 to give structure **10**) were absent, but there was a singlet of intensity 2H at 4.77 ppm. These results clearly indicated that the reaction between ethyl chloroacetate and the indolizyl anion led to 5-chloroacetyl-indolizine **9**; this was confirmed by elemental analysis data.

### 2.5. Conclusions

Indolizyl lithium derivatives **A** and **B** react with various bielectrophiles giving 1,4-keto acids (with succinic and phtalic anhidrides), 1,2-diketone or 1,2-keto acid (with oxallyl chloride) and 1,2-hydroxyacid (with ethyl pyruvate). However, with α-halocarbonyl compounds, they react in different ways, forming the products of selective bromination at C-5 (with α-bromo ketones and esters of α-bromo acids) and 5-chloroacetyl indolizines (with esters of α-chloroacetic acid). No spontaneous formation of a tricyclic structure was observed in these cases.

## 3. Experimental Section

### 3.1. General Information

All experiments involving air-sensitive compounds were performed with freshly distilled solvents under anhydrous conditions in oven-dry glassware with rubber septa under the pressure of argon using standard Schlenk techniques. ^1^H- and ^13^C-NMR spectra were recorded on a Bruker WP-400 spectrometer (operating frequency 400 MHz) (Bruker Ltd., Billerica, MA, USA) using commercially available DMSO-*d*_6_ and CDCl_3_ with TMS as internal standard. Mass-spectra were recorded on a Kratos MS-30 instrument (70 eV) (Kratos Analytical Ltd/Shimadzu group, Manchester, UK). Melting points were determined on Electrotermal IA 9000 (Cole-Parmer, Staffordshire, UK). The reaction progress was monitored by means of thin-layer chromatography (TLC) on aluminum foil plates, covered with silica gel 90-120 F254 “Sorbfil” using either UV light and Ehrlich′s reagent as a visualizing agent. Product purifications were done by flash chromatography using 230−400 mesh silica gel (Merck KGaA, Darmstadt, Germany).

### 3.2. Synthesis

*4-(2-Phenylindolizin-5-yl)-4-oxobutanoic acid* (**2a**). The solution of 0.5 g (2.6 mmol) 2-phenylindolizine (**1a**) and 0.36 mL (4.0 mmol) tetramethylethylenediamine (TMEDA) in 150 mL THF was degassed, filled with argon and cooled to −80 °C. Thereto, the solution of 1.8 mL (4 mmol) of 2.24 M *n*-BuLi in hexane was added dropwise. The mixture was allowed to spontaneously warm up to −10 °C and stirred at this temperature for 2 h. Then solution of 0.4 g (4.0 mmol) of succinic anhydrid in 10 mL THF was added quickly at −90 ° C. The reaction mixture was allowed to warm to room temperature and stand for several hours, and then it was poured into a stirred mixture of 100 mL CH_2_Cl_2_ and 100 mL of saturated aqueous NH_4_Cl solution. The organic layer was separated, washed with water and dried over Na_2_SO_4_. The solvent was removed in vacuo. The crude product was placed on silica gel and purified by preparative chromatography (eluent–CHCl_3_:MeOH = 10:1). Yield: 0.4 g (53%); yellow powder; m.p. 204–205 °C, ^1^H-NMR (DMSO-*d*_6_): 12.25 (1H, bs, OH), 9.27 (1H, s, H-3), 7.89–7.86 (2H, m, H-6, H-8), 7.76–7.74 (2H, m, Ph), 7.43–7.39 (2H, m, Ph), 7.29–7.25 (1H, m, Ph), 7.12 (1H, s, H-1), 6.90 (1H, d, *J* = 7.5 Hz, H-7), 3.38 (2H, m, CH_2_), 2.67–2.64 (2H, m, CH_2_); ^13^C-NMR (DMSO-*d*_6_) 188.06 (CO), 173.82 (CO_2_H), 141.56 (C-2), 137.44 (C-5), 134.34 (Ph), 129.86 (C-9),128.86 (C-Ph), 126.79 (C-Ph), 125.75 (C-Ph), 120.72 (C-8), 120.68 (C-7), 115.41 (C-3), 112.99 (C-6), 99.48 (C-1), 32.99 (CH_2_), 28.19 (CH_2_); C_18_H_15_NO_3_·0.5H_2_O, calcd., %: C 71.51; H 5.33; N 4.63; found, %: C 71.23; H, 5.55; N 4.46.

*4-(2-tert-Butylindolizin-5-yl)-4-oxobutanoic acid* (**2b**). The solution of 0.45 g (2.6 mmol) 2-*tert*-butylindolizine (**1b**) and 0.36 mL (4.0 mmol) TMEDA in 70 mL THF was degassed, filled with argon and cooled to −80 °C. Thereto, the solution of 1.8 mL (4 mmol) of 2.24 M *n*-BuLi in hexane was added dropwise. The mixture was allowed to spontaneously warm up to −10 °C and stirred at this temperature for 2 h. Then, the solution of 0.4 g (4.0 mmol) of succinic anhydrid in 10 mL THF was added quickly at −90 °C. The reaction mixture was allowed to warm to room temperature and stand for several hours, and then was poured into a stirred mixture of 100 mL CH_2_Cl_2_ and 100 mL of saturated aqueous NH_4_Cl solution. The organic layer was separated, washed with water and dried over Na_2_SO_4_. The solvent was removed in vacuo. The crude product was placed on silica gel and purified by preparative chromatography (eluent–CHCl_3_: MeOH = 10:1). Yield: 0.52 g (73%); yellow crystals; m.p. = 161–164 °C; ^1^H-NMR (DMSO-*d*_6_): 8.79 (1H, s, H-3), 7.83–7.78 (2H, m, H-6, H-8), 6.85–6.81 (1H, m, H-7), 6.67 (1H, s, H-1), 3.36–3.32 (2H, m, CH_2_), 2.65-2.62 (2H, m, CH_2_), 1.33 (9H, s, *t*-Bu); ^13^C-NMR (DMSO-*d*_6_) 190.73 (CO), 170.80 (CO_2_H), 141.82 (C-2), 134.16 (C-5), 128.83(C-9), 124.78 (C-8), 120.11 (C-7), 114.33 (C-3), 112.51 (C-6), 99.91 (C-1), 32.89 (CH_2_), 28.15 (CH_2_), 31.66 (*t*-Bu), 30.80 (*t*-Bu); MS (*m*/*z*): 273 (M^+^, 100), 259 (30), 172 (55); X-ray data, see Figure 1, Table 1.

*2-[(2-Phenylindolizin-5-yl)carbonyl]benzoic acid* (**3a**). Synthesized according to the general procedure with phtalic anhydride. Yield: 0.443 g (50%); red powder; m.p. = 129–131 °C; ^1^H-NMR (DMSO-*d*_6_): 9.28 (1H, s, H-3), 8.02 (1H, d, *J* = 7.0 Hz, H-6), 7.83–7.79 (3H, m, Ar), 7.77–7.73 (1H, m, Ar), 7.70–7.66 (2H, m, Ar), 7.60 (1H, d, *J* = 7.0 Hz, H-8), 7.47–7.43 (2H, m, Ar), 7.30 (2H, m, H-7), 7.16 (1H, s, H-1), 3.36 (1H, s, CO_2_H); ^13^C-NMR (DMSO-*d*_6_) 191.58 (CO), 170.20 (CO_2_H), 140.81 (C-2), 135.00 (C-5), 134.43 (Ph), 129.84 (C-9), 129.00 (C-Ph), 126.90 (C-Ph), 125.82 (C-Ph), 124.37 (C-8), 121.82 (C-7), 115.65 (C-3), 112.93 (C-6), 99.33 (C-1), 141.49 (Ar), 128.53 (Ar), 129.93 (Ar), 129.77 (Ar), 130.17 (Ar), 131.16 (Ar); MS (*m*/*z*): 341 (M^+^, 60), 312 (30), 296 (85), 268 (100); C_22_H_15_NO_3_·2H_2_O, calcd., %: C 70.02; H 5.07; N 3.71; found, %: C 69.75; H 4.79; N 3.70.

*2-[(2-tert-Butylindolizin-5-yl)carbonyl]benzoic acid* (**3b**). Prepared similarly. Yield: 0.47 g (56%); red powder; m.p. = 113–115 °C; ^1^H-NMR (DMSO-*d*_6_): 8.94 (1H, s H-3), 7.96–7.93 (1H, m, H-6), 7.56–7.54 (1H, m, Ar), 7.50–7.48 (2H, m, Ar), 7.36–7.35 (1H, m, Ar), 6.70–6.68 (1H, m, H-8), 6.61 (1H, s H-1), 6.43–6.40 (1H, m, H-7), 1.39 (9H, s t-Bu); ^13^C-NMR (DMSO-*d*_6_) 191.17 (CO), 170.26 (CO_2_H), 143.11 (C-2), 134.53 (C-5), 129.54 (C-9), 124.74 (C-8), 120.23 (C-7), 114.16 (C-3), 113.62 (C-6), 100.33 (C-1), 142.29 (Ar), 128.21 (Ar), 132.85 (Ar), 128.23 (Ar), 130.31 (Ar), 131.03 (Ar), 31.91 (*t*-Bu), 31.29 (*t*-Bu); 3C_20_H_19_NO_3_·4H_2_O, calcd., %: C 69.55, H 6.32, N 4.06, found, %: C 69.22, H 6.05, N 4.00.

*Oxo(2-phenylindolizin-5-yl)acetyl chloride* (**4a**). Prepared using 5-fold excess of oxallyl chloride. The resulting precipitate was filtered off and dried in vacuo. The product was used without further purification. Yield: 0.8 g; red powder; mixture of oxo(2-phenylindolizin-5-yl)acetyl chloride with an unknown amount of inorganic lithium salts; ^1^H-NMR (DMSO-*d_6_*): 8.10 (1H, m, H-3), 7.95 (1H, m, H-6), 7.86 (1H, m, Ph), 7.76 (2H, m, Ph), 7.43 (5H, m, Ph, Ar),7.06 (1H, m, Ar); MS (*m*/*z*): 440 (30), 283 (40).

*1,2-di(2-tert-Butylindolizin-5-yl)ethane-1,2-dione* (**5b**). Synthesized according to the general procedure with the ratio indolizine–oxallyl chloride 2:1. Yield: 0.2 g (50%); red needles; m.p. = 207–210 °C; ^1^H-NMR (DMSO-*d*_6_): 8.96 (1H, s, H-3), 7.98 (1H, d, *J* = 8.4 Hz, H-6), 7.58 (1H, d, *J* = 7.2 Hz, H-8), 6.90 (1H, s, H-1), 6.83 (1H, dd, *J* = 7.2 Hz, *J* = 8.4 Hz, H-7), 1.39 (9H, c, *t*-Bu); ^13^C-NMR (DMSO-*d*_6_): 187.75 (CO), 143.28 (C-2), 134.40 (C-5), 127.15(C-9), 127.22 (C-8), 125.65 (C-7), 114.76 (C-3), 113.84 (C-6), 102.04 (C-1), 31.91 (*t*-Bu), 31.29 (*t*-Bu). X-ray data, see Figure 2, Table 1.

*Methyl (2-tert-butylindolizin-5-yl)(oxo)acetate* (**6b**). Synthesized from **1b** using reverse order in mixing of reactants. Indolizyl lithium **B** was added to oxallyl chloride, the reaction mixture was allowed to warm to room temperature and 20 mL 0.2 M solution of MeONa in MeOH was added dropwise. The mixture was then evaporated to dryness, extracted with CHCl_3_, and evaporated again. The resulting solid was purified by preparative chromatography (silica gel, eluent–hexane, then –CHCl_3_:MeOH = 10:1) followed by recrystallization (hexane–acetone). Yield: 0.2 g (30%); red needles; m.p. = 225−228 °C; ^1^H-NMR (DMSO-*d*_6_): 8.94 (1H, s, H-3), 7.96 (1H, d, *J* = 8.3 Hz, H-6), 7.56 (1H, d, *J* = 7.2 Hz, H-8), 6.87 (1H, m, H-1), 6.82 (1H, dd, *J* = 7.2 Hz, *J* = 8.3 Hz, H-7), 2.07 (3H, s, OMe), 1.38 (9H, s, *t*-Bu). C_15_H_17_NO_3_, calcd., %: C 69.48, H 6.61, N 5.40, found, %: C 68.99, H 6.64, N 5.36.

*Ethyl 2-(2-tert-butylindolizin-5-yl)-2-hydroxypropanoate* (**7**). Synthesized according to the general procedure with ethyl 2-oxopropanoate. Yield: 0.56 g (75%); clear oil; ^1^H-NMR (DMSO-*d*_6_): 7.85 (1H, s, H-3), 7.63 (2H, m, Ph), 7.47–7.39 (3H, m, Ph), 7.27–7.23 (1H, m, H-8), 6.86 (1H, s, H-1), 6.78–6.77 (2H, m, H-6, H-7), 6.45 (1H, s, OH), 4.16–4.03 (2H, m, CH_2_), 1.86 (3H, c, CH_3_), 1.04 (3H, m, CH_2_CH_3_); ^13^C-NMR (DMSO-*d*_6_): 175.71 (CO_2_Et), 141.36 (C-2), 134.30 (C-5), 133.89 (C-9), 108.68 (C-8), 118.96 (C-7), 115.41 (C-3), 108.56 (C-6), 97.34 (C-1), 31.84 (*t*-Bu), 30.95 (*t*-Bu), 74.82 (COH), 62.74 (OEt) 25.30 (CH_3_), 13.87 (OEt); 2C_19_H_19_NO_3_·H_2_O, calcd.,%: C 71.68, H 6.33, N 4.40; found,%: C 71.90, H, 6.43, N 4.22.

*5-Bromo-2-phenylindolizine* (**8a**). Synthesis with phenacyl bromide. Yield: 0.42 g (60%); yellow crystals; m.p. = 85–87 °C; ^1^H-NMR (CDCl_3_): 7.86 (1H, s, H-3), 7.71–7.69 (2H, m, Ph), 7.43–7.37 (3H, m, Ph), 7.29 (1H, d, *J* = 7.7 Hz, H-6), 6.89 (1H, s, H-1), 6.78 (1H, d, *J* = 7.9 Hz, H-7), 6.60–6.56 (1H, m, H-8). Identical to the sample obtained by using (BrCF_2_)_2_ [12].

*5-Bromo-2-tert-butylindolizine* (**8b**). Prepared similarly. Yield: 0.47 g (72%); pale yellow oil; (CDCl_3_): 7.41 (1H, s, H-3), 7.30 (1H, d, *J* = 8.7 Hz, H-6), 6.71 (1H, d, *J* = 7.0 Hz, H-8), 6.54 (1H, m, H-7), 6.51 (1H, s, H-1), 1.37 (9H, c, *t*-Bu). Identical to the sample obtained by using (BrCF_2_)_2_ [12].

*5-Bromo-2-tert-butylindolizine* (**8b**). From the reaction with ethyl bromoacetate. Yield: 0.46 g (72%). Identical to the previous sample. 

*2-Chloro-1-(2-phenilindolizin-5-yl)ethanone* (**9**). Synthesized according to the general procedure with chloroacetic ester. Yield: 0.21 g (30%); red powder; m.p. = 105–107 °C; ^1^H-NMR (CDCl_3_): 9.44 (1H, s, H-3), 7.79–7,75 (3H, m, Ph), 7.57 (1H, d, *J* = 6 Hz, H-6), 7.46–7.43 (2H, m, Ph), 7.32 (1H, d, *J* = 6 Hz, 8-H), 7.05 (1H, s, H-1), 6.80 (1H, m, H-7), 4.77 (2H, s, CH_2_); C_16_H_12_ClNO, calcd., %: C 71.25; H 4.48; N 5.19; found, %: C 71.45, H 4.59; N 5.20.

### 3.3. X-ray Diffraction Study of Compounds ***2b*** and ***5b***

The parameters of the unit cell of compound **2b** were defined and refined on 8289 reflections in the range of angles 2.70–29.96°. Primary processing of experimental data was carried out by *APEX2* and *SAINT-Plus* programs [18]. All subsequent calculations (solution and refinement of the structures) were made using *SHELX97* programs [19]. The parameters of the unit cell of compound **5b** were defined and refined on the 12,101 reflections in the range of angles и 2.41–25.24°. Primary processing of experimental data was carried out with the program *X-AREA* [20]. All subsequent calculations (solution and refinement of the structures) were made using *SHELX97* programs [19]. Molecular representation for **2b** and **5b** was made using the program *ORTEP-3* [21]. Editing of CIF files was performed using *WinGX* program [21]. Crystal structures were refined in the anisotropic approximation for all non-hydrogen atoms. The hydrogen atoms were placed in calculated positions and refined the model rider-atom—*U*_iso_ (H) = (1.2–1.5) *U*_eq_ (C). The hydrogen atom in the hydroxy group of **2b** was conducted independently in the isotropic approximation. Crystal data and structure refinement for structures **2b** and **5b** are given in Table 1. The structural information on the investigated compounds is deposited in the Cambridge Structural Database [22] (CCDC 1010131 (**2b**), CCDC 1010132 (**5b**)).

## Figures and Tables

**Table 1:** Crystal data and structure refinement for structures **2b** and **5b**.

Identification Compound Code	2b	5b
Empirical formula	C_16_H_19_NO_3_	C_26_H_28_N_2_O_2_
Formula weight	273.32	400.50
Temperature, K	100(2)	295(2)
Wavelength, Å	0.71073	0.71073
Crystal system	Orthorhombic	Monoclinic
Space group	*Pbca*	*P*2_1_/*c*
a, Å	8.4621(5)	14.219(2)
b, Å	10.9254(6)	9.9954(13)
c, Å	30.2222(18)	16.642(3)
α, deg	90	90
β, deg	90	107.735(13)
γ, deg	90	90
Volume, Å^3^	2794.1(3)	2252.8(6)
*Z*	8	4
Density (calculated), Mg/m^3^	1.299	1.181
Absorption coefficient, mm^−1^	0.090	0.075
*F*(000)	1168	856
Crystal size, mm	0.30 × 0.15 × 0.15	0.10 × 0.10 × 0.10
Theta range for data collection, deg.	2.70–30.02	2.41–25.24
Index ranges	−11 ≤ *h* ≤ 11, −15 ≤ *k* ≤ 15, −42 ≤ *l* ≤ 42	−17 ≤ *h* ≤ 8, −12 ≤ *k*≤ 12, −20 ≤ *l* ≤ 20
Reflections collected	30,104	34,200
Independent reflections [*R*_int_]	4074 [0.0411]	11014 [0.0261]
Max. and min. transmission	------------------------	---------------------------
Data/restraints/parameters	4074/0/188	34,200/0/274
Goodness-of-fit on *F*^2^	1.053	0.843
*R*_1_/w*R*_2_, *I* > 2δ(*I*)	0.0537/0.1382	0.0448/0.0841
*R*_1_/w*R*_2_, all data	0.0682/0.153	0.1694/0.1156
Δ*р*_max_/Δ*р*_min_, e/Å^3^	0.512/−0.242	0.203/−0.178
Extinction coefficient	------------------------	0.0053(5)
Diffractometer model	Bruker APEX-II CCD	STADI-VARY Pilatus-100K

## References

[1] Liu B., Wang Z.-J., Wu N., Li M., You J., Lan J. (2012). Discovery of a full-color-tunable fluorescent core framework through direct C H (hetero) arylation of *N*-heterocycles. Chem. Eur. J..

[2] Rotaru A.V., Druta I., Oeser T., Mϋller T.J.J. (2005). A novel coupling 1,3-dipolar cycloaddition sequence as a three-component approach to highly fluorescent indolizines. Helv. Chim. Acta.

[3] Kim E., Lee S., Park S.B. (2012). A Seoul-fluor-based bioprobe for lipid droplets and its application in image-based high throughput screening. Chem. Commun..

[4] Singh G.S., Mmatli E.E. (2011). Recent progress in synthesis and bioactivity studies of indolizines. Eur. J. Med. Chem..

[5] Vemula V.R., Vurukonda S., Bairi C.K. (2011). Indolizine derivatives: Recent advances and potential pharmacological activities. Int. J. Pharm. Sci. Rev. Res..

[6] Huang W., Zuo T., Luo X., Jin H., Liu Z., Yang Z., Yu X., Zhang L., Zhang L. (2013). Indolizine derivatives as HIV-1 VIF-elongin C interaction inhibitors. Chem. Biol. Drug Des..

[7] Flitsch W., Katritzky A., Rees C.W. (1984). Pyrroles with fused six-membered heterocyclic rings: A-fused. Comprehensive Heterocyclic Chemistry.

[8] Swinborne P.-J., Hunt J.H., Klinkert G. (1978). Advances in indolizine chemistry. Adv. Heterocycl. Chem..

[9] Prostakov N.S., Baktibaev O.B. (1975). Indolizines. Usp. Khim..

[10] Renard M., Gubin J. (1992). Metallation of 2-Phenylindolizine. Tetrahedron Lett..

[11] Kuznetsov A.G., Bush A.A., Rybakov V.B., Babaev E.V. (2005). An Improved Synthesis of Some 5-Substituted Indolizines Using Regiospecific Lithiation. Molecules.

[12] Kuznetsov A.G., Bush A.A., Babaev E.V. (2007). Synthesis and Reactivity of 5-Br(I)-Indolizines and their Parallel Cross-coupling Reactions. Tetrahedron.

[13] Shadrin I.A., Rzhevskii S.A., Rybakov V.B., Babaev E.V. (2015). Sonogashira Reaction of the Indolizine Ring. Synthesis.

[14] Babaev E.V., Torocheshnikov V.N., Bobrovsky S.I. (1995). NMR spectra of indolizines and their sigma-complexes. Chem. Heterocycl. Comp..

[15] Fraser M., Mc Kenzie S., Reid D.H. (1966). Nuclear magnetic resonance. Part IV. The protonation of indolizines. J. Chem. Soc. (B).

[16] Armarego W.L.F. (1966). C-1 and C-3 protonation of indolizines. J. Chem. Soc. (B).

[17] Windgassen R.J., Saunders W.H., Boekelheide V. (1959). Cyclazines. A new class of aromatic heterocycles. J. Am. Chem. Soc..

[18] Bruker (2012). APEX2 and SAINT-Plus.

[19] Sheldrick G.M. (2008). A short history of SHELX. Acta Crystallogr..

[20] Stoe & Cie (2012). X-AREA.

[21] Farrugia L.J. (2012). WinGX and ORTEP for WI: An update. J. Appl. Crystallogr..

[22] Groom C.R., Allen F.H. (2014). The Cambridge Structural Database in Retrospect and Prospect. Angew. Chem. Int. Ed..

